# Aging modulates the immunosuppressive, polarizing and metabolic functions of blood-derived myeloid-derived suppressor cells (MDSCs)

**DOI:** 10.1186/s12979-025-00524-w

**Published:** 2025-07-08

**Authors:** Emma Keltsch, Jennifer Greiner, Lena Wahl, Ingrid Knape, Daniel Tews, Michael Denkinger, Klaus-Michael Debatin, Gudrun Strauss

**Affiliations:** 1 Department of Pediatrics and Adolescent Medicine, University Medical Center Ulm, Eythst. 24, 89075 Ulm, Germany; 2 Institute for Geriatric Research, Ulm University and AGAPLESION Bethesda Clinic, Geriatric Center Ulm, Zollernring 26, 89073 Ulm, Germany; 3 German Center for Child and Adolescent Health (DZKJ), partner site Ulm, Ulm, Germany

**Keywords:** Immunosenescence, Myeloid-derived suppressor cells, T cell immunity

## Abstract

**Background:**

Immunosenescence describes the gradual remodeling of immune responses, leading to disturbed immune homeostasis and increased susceptibility of older adults for infections, neoplasia and autoimmunity. Decline in cellular immunity is associated with intrinsic changes in the T cell compartment, but can be further pushed by age-related changes in cells regulating T cell immunity. Myeloid-derived suppressor cells (MDSCs) are potent inhibitors of T cell activation and function, whose induction requires chronic inflammation. Since aging is associated with low grade inflammation (inflammaging) and increased myelopoiesis, age-induced changes in MDSC induction and function in relation to T cell immunity were analyzed.

**Results:**

MDSC numbers and functions were compared between “healthy” young and old adults, who were negatively diagnosed for severe acute and chronic diseases known to induce MDSC accumulation. MDSCs were either isolated from peripheral blood or generated in vitro from blood-derived CD14 cells. Aging was associated with significantly increased MDSC numbers in the monocytic- (M-) and polymorphonuclear (PMN-) MDSC subpopulations. MDSCs could be induced more efficiently from CD14 cells of old donors and these MDSCs inhibited CD3/28-induced T cell proliferation significantly better than MDSCs induced from young donors. Serum factors of old donors supported MDSC induction comparable to serum factors from young donors, but increased immunosuppressive activity of MDSCs was only achieved by serum from old donors. Elevated immunosuppressive activity of MDSCs from old donors was associated with major metabolic changes and increased intracellular levels of neutral and oxidized lipids known to promote immunosuppressive functions. Independent of age, MDSC-mediated suppression of T cell proliferation required direct MDSC– T cell contact. Besides their increased ability to inhibit activation-induced T cell proliferation, MDSCs from old donors strongly shift the immune response towards Th2 immunity and might thereby further contribute to impaired cell-mediated immunity during aging.

**Conclusions:**

These results indicate that immunosenescence of innate immunity comprises accumulation and functional changes in the MDSC compartment, which directly impacts T cell functions and contribute to age-associated impaired T cell immunity. Targeting MDSCs during aging might help to maintain functional T cell responses and increase the chance of healthy aging.

**Supplementary Information:**

The online version contains supplementary material available at 10.1186/s12979-025-00524-w.

## Background

Aging characterizes a decline of function in all organ systems during adulthood. Age-related changes of innate and adaptive immune functions are defined as immunosenescence, a process induced by four major hallmarks: thymic involution, changes in hematopoiesis and metabolism and increased inflammation [1]. Already in early childhood, thymic involution initiates aging of the cellular immune response by a decline in naïve T cell export and the concomitant increase of memory T cells. As a consequence, the diversity of the TCR repertoire is constantly reduced during lifetime, impairing the effectiveness of the T cell response against new antigens and vaccinations and at the same time increasing the susceptibility to infections and cancer [2]. Mutual relationship of innate and adaptive immune cells implies that age-related modulations in cellular immunity are not only attributed to intrinsic T cell changes but also to cells regulating T cell functions.

Myeloid-derived suppressor cells (MDSCs) are a heterogenous population of immature myeloid cells which modulate innate and adaptive immune responses. MDSCs are grouped into two major subpopulations: monocytic (M-) MDSCs and polymorphonuclear (PMN-) MDSCs, indicating their close relation to mature monocytes and granulocytes. Phenotypic characterization defines human M-MDSCs as CD33^+^CD11b^+^CD14^+^HLA-DR^−/low^ and PMN-MDSCs as CD33^+^CD11b^+^CD66b^+^HLA-DR^−/low^ [3, 4]. Although MDSCs can modulate the function of mostly all cells of the innate and adaptive immunity, T cells are the major targets of MDSCs [5]; e.g. impaired anti-tumor T cell responses in solid cancers mostly go along with significantly elevated MDSC numbers. MDSC accumulation, however, is not only driven by cancers, but generally increases during chronic inflammation such as autoimmunity, trauma, sepsis or viral infections [6].

Since low grade chronic inflammation (inflammaging) is considered as one of the hallmarks of aging, MDSC levels are likely to be changed during lifetime. Early studies by Verschoor et al. [7] reported significantly elevated MDSCs levels in peripheral blood of frail elderly with a median age of 87 yrs compared to healthy adults with a median age of 32 yrs. A further increase in MDSC numbers occurred in old persons with a history of cancer. While Verschoor et al. did not distinguish between the different MDSC subpopulations, studies by Alves et al. showed accumulation of only PMN-MDSCs in people over 80 yrs compared to young people (20–30 yrs) [8]. Currently, there is very little knowledge about the influence of age per se on MDSC expansion and function, since it is not an easy solvable problem to distinguish between MDSC induction due to age or age-related diseases. Studies in mice, where the influence of external factors can be excluded and the development of age-related comorbidities can easily be scored, already give an indication that MDSC numbers increase with age [9, 10].

Two major age-specific conditions presumably contribute to MDSC accumulation. First, overtly healthy older adults commonly exhibit modestly heightened levels of pro-inflammatory mediators in the blood (inflammaging). In old individuals, different cellular sources such as senescent immune cells and fibroblasts are in a tonically activated state, producing elevated levels of inflammatory mediators such as TNFα, IL-1α, IL-1β, IL-6 and PGE_2_ in the absence of infections [11–14]. Whether inflammatory mediators cause MDSC induction and expansion only by themselves or in concert with a dysregulated hematopoiesis, the second age-related condition supporting MDSC expansion, is not finally clarified. With age the ability of hematopoietic stem and progenitor cells (HSPCs) to self-renew, engraft and differentiate decreases and hematopoiesis is shifted towards the increased production of myeloid cells at the expense of lymphoid cells, a process called myeloid skewing. Intrinsic changes within the HSPCs and accumulation of HSPC mutations lead to increased expression of myeloid-specific genes, altered proliferation and cell cycle kinetics and increased in vivo myelopoiesis [15, 16].

Whether increased MDSC numbers translate into age-related molecular and functional changes is not yet well defined. Old mice do not only exhibit an increase in MDSC numbers but also an improved immunosuppressivity [9, 17]. To the best of our knowledge, detailed molecular and functional comparisons of young and old MDSCs in humans are rare. Comparison of pediatric and elderly in vitro-generated MDSCs derived from bone marrow (BM) indicate no age-related changes in the immunosuppressive ability [18]. However, many age-related human T cell features such as a reduced activation capacity and cytokine secretion, an exhausted phenotype or the decline of the Th1/Th2 balance [2], are also T cell characteristics associated with the presence of MDSCs in other diseases such as cancer and autoimmunity [19, 20].

Therefore, in this study, we did a comprehensive comparison of MDSC numbers and functions in young and old non-frail individuals. To our knowledge, we can show for the first time an increase of MDSCs in old persons, which is not related to the development of a chronic disease such as cancer, autoimmunity, neurodegeneration or cardiovascular disease. MDSCs derived from blood CD14 cells of old persons exhibit an increased immunosuppressive potential, turn the balance towards Th2 immune responses and upregulate metabolic pathways associated with improved MDSC functionality. Our findings suggest that elevated MDSC numbers during aging might further compromise the physiological functions of T cells in old individuals and therefore support the development of age-associated diseases.

## Materials and methods

### Patient information

Peripheral EDTA blood (PB) was taken from young donors between 20 and 25 years or old donors between 72 and 84 years. Donors of PB were all females. Exclusion criteria were cancer, acute infections, autoimmune, neurodegenerative and severe cardiovascular diseases. Viral status concerning e.g. CMV, EBV or HSV was not analyzed. Informed consent was obtained from all participants, in compliance with all the relevant national regulations, institutional policies and in accordance the tenets of the Helsinki Declaration, and has been approved by the local Ethical Committee. Buffy Coats (BC) were obtained either from young (18–22 yrs) or old (61–70 yrs) donors. Gender distribution: young BC donors: 80% females, 20% males; old BC donors: 33% females, 67% males. Age of donors for PB and BC differs, since BC are only prepared from persons until the age of 70.

### Cell culture, isolation of PBMCs, T cells and CD14 cells

PBMCs were isolated from PB or BCs by density centrifugation on BioColl^®^ gradient solution (Bio&Sell). T cells were isolated from BCs by RosetteSepHLA T cell enrichment cocktail (Stemcell Technologies). CD14 cells were isolated by negative selection using RosetteSep™ human monocyte enrichment cocktail. Purity of T cells and CD14 cells was confirmed by flow cytometry by staining for CD2 and CD3 and CD14 expression gaining a T cell purity of 94–99% and CD14 purity of > 74%. T cells were cultured in complete RPMI 1640 medium (Life Technologies) supplemented with 2% FCS (Lonza), 2 mM L-glutamine, and 100U/ml penicillin-streptomycin at 37 °C in a humidified atmosphere containing 7,5% CO_2_, while CD14 cells were cultured in complete medium containing 10% FCS. Proliferation experiments were carried out in complete medium containing 5% FCS.

### Differentiation of blood-derived MDSCs

CD14 cells isolated either from PB or BCs were cultured with 5 × 10^5^/ml in complete medium (10% FCS) in the presence of human IL-6 (20ng/ml) and GM-CSF (20ng/ml) (Peprotech) for 5 days. IL-6 (20ng/ml) and GM-CSF (20ng/ml) were re-added to the cultures on day 2. Efficiency of MDSC differentiation was defined on day 5 by down-regulation of HLA-DR by flow cytometry and inhibition of T cell activation. Cells were used if HLA-DR expression was downregulated on 50–70% of the cells compared to unstimulated CD14 cells. MDSCs derived from CD14 cells of PB were named PB-MDSCs, while MDSCs derived from BCs were named BC-MDSCs.

### CFSE labeling and T cell activation in the presence of blood-derived MDSCs

2 × 10^6^ T cells were labeled with 5 µM CFSE (ThermoFisher Scientific) at 37 °C for 10 min, immediately washed with ice-cold PBS-5%FCS, and subsequently used for proliferation assays. 2 × 10^5^/well CFSE-labeled T cells were activated with plate-bound CD3 (0.1 µg/well) and CD28 antibodies (0.1 µg/well) in the presence of blood-derived MDSCs at different T cell: MDSC ratios or in the absence of blood-derived MDSCs. Transwell assays were performed in 24 well plates with inserts of 0.4 μm (Falcon). 1.2 × 10^6^ T cells / well were activated with plate-bound CD3 (0.6 µg/well) and CD28 antibodies (0.6 µg/well). Blood-derived MDSCs were added at a T cell: MDSC ratio of 1:1 either directly to the T cells or to the transwell insert. T cell proliferation assays were harvested at day 5 and T cell proliferation was defined by CFSE dilution by staining cells for CD2. Percentage of specific proliferation = (% stimulus-induced proliferating T cells−% proliferating T cells in medium alone)/(100−% proliferating T cells in medium alone)×100 was calculated. Likewise, the proliferation and division index was defined with FlowJo software (10.8.1) [21].

### Cytokine analysis in serum and cell culture supernatants

Blood was collected in S-Monovette^®^Serum (Sarstedt). After allowing clotting for 30 min at RT, blood was centrifuged and the supernatant collected. Serum samples were aliquoted and immediately stored at -80 °C. Likewise, supernatants from proliferation assays were collected at day 4 and immediately stored at -80 °C. Serum and supernatant factors were analyzed by ProcartaPlex Multiplex immunoassays (Thermo Fisher Scientific) on a BIO-RAD Bioplex.

### Flow cytometry

A total of 5–10 × 10^5^ cells were stained in FACS-medium (PBS − 10% FCS − 0.2% NaN_3_). 7-amino-actinomycin-D (7-AAD, Sigma-Aldrich) positivity was used for exclusion of dead cells. Antibodies used are specified in Supplementary Table 1. MDSCs were stained with CD14-PacificBlue followed by staining with 1.2µM BODIPY 493/503 or 2.5µM BODIPY 581/591 C11 (ThermoFisher Scientific) to detect neutral or oxidized lipids. Flow cytometry samples were measured on LSR II flow cytometer (BD Bioscience). Gating of cell populations was done by hand.

### RNA preparation and quantitative reverse-transcription polymerase chain reaction (qRT-PCR)

RNA was isolated and complementary DNA (cDNA) was synthesized as previously described [22] and qRT-PCR was performed with a CFX Connect™ Real-Time PCR Detection System (Bio-Rad) using a LightCyler FastStart DNA Master PLUS SYBR Green I Kit (Roche Diagnostics). The qRT-PCR results were normalized using human HPRT1 as house-keeping gene. Primer sets (Biomers.net GmbH, Ulm, Germany) used are listed in Supplementary Table 2.

### RNA-seq analysis

cDNA libraries were constructed and sequenced by the Beijing Genomics Institute (BGI, Beijing, China) using the DNBSEQ platform. Raw reads were filtered with SOAPnuke and clean reads were aligned to the reference genome (Homo_sapiens, NCBI, GCF_000001405.39_GRCh38.p13) using HISAT2. Bowtie2 was applied to map the reads to the gene set. Data analysis was conducted on the Dr. Tom online platform (https://eu-biosys.bgi.com) provided by BGI. Samples were excluded from analysis if intragroup correlations were < 0.9. Differential expression gene (DEG) analysis was performed using the DESeq2 package (|log2(FC)| ≥ 0.5; q < 0.05). Gene Ontology (GO) analysis of DEGs was performed with a significance threshold of *p* < 0.05. Gene Set Enrichment Analysis (GSEA) employed the Hallmark gene set, with normalized enrichment score (NES)| > 1 or < -1, *p* < 0.05 and false discovery rate (FDR) < 0.25). Principal component analysis (PCA) was performed using iDEP.96 (http://bioinformatics.sdstate.edu/idep96/). GraphPad Prism 10 was used for data visualization. Raw and processed RNA-seq data have been deposited in the NCBI Gene Expression Omnibus (GEO) under accession number GSE279455.

### Statistical analysis

Data was tested for normal distribution using the D’Agostino-Pearson and Shapiro-Wilk normality tests. For normally distributed data, an unpaired Student’s t-test was used for two-group comparisons. Multiple groups were analyzed with either multiple unpaired t-tests corrected by the Holm- Šídák method or two-way ANOVA with Šídák or Tukey’s multiple comparisons test. Multiple groups were analyzed using multiple unpaired t-tests with Holm-Šídák correction, ordinary one-way ANOVA, or two-way ANOVA followed by Šídák’s or Tukey’s multiple comparisons test. For nonparametric data, the Mann–Whitney test was used for two-group comparisons, and the Kruskal-Wallis test was used for comparisons involving three or more groups. Correlations were assessed using Spearman’s rank correlation with a two-tailed p-value, followed by simple linear regression. Statistical tests were performed with GraphPad Prism 10.

## Results

### Aging is associated with MDSC expansion in healthy donors

**Fig. 1:**
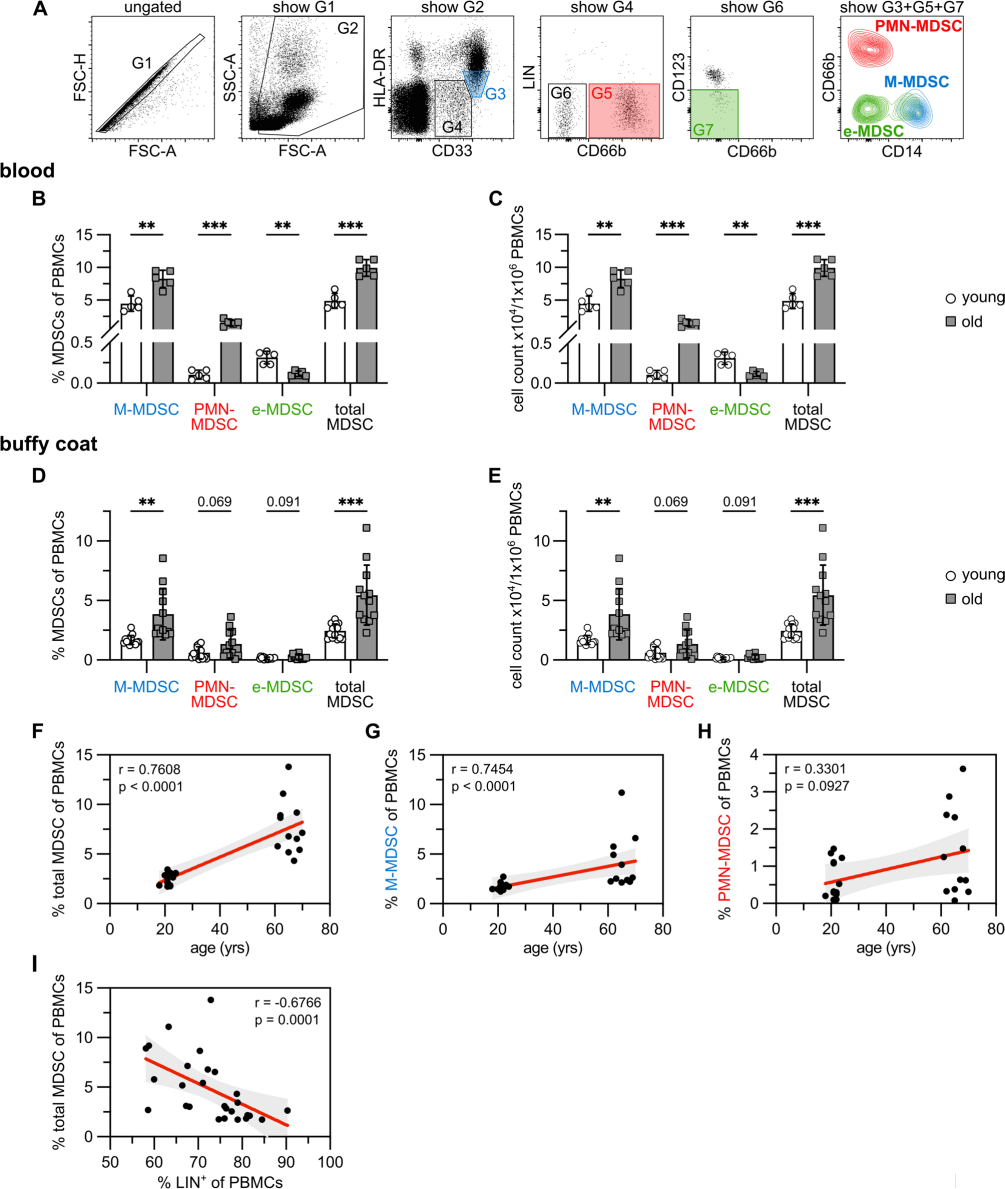
Aging is associated with MDSC accumulation. **A**: Representative gating scheme for MDSCs from PBMCs isolated from peripheral blood or buffy coats. Graphs are derived from PBMCs of peripheral blood. **B-E**: PBMCs were isolated from peripheral blood (**B, C**) or buffy coats (**D, E**) of young and old donors and stained for MDSCs as shown in A. Percentage (**B, D**) and absolute numbers (**C, E**) of total-, M-, PMN- and e-MDSCs were calculated. **F-H**: Correlation of percentage of total (**F**), M- (**G**) and PMN- (**H**) MDSCs in buffy coats with age. **I**: Correlation of percentage of total MDSCs with Lin+ (CD3+, CD19+, CD20+, CD56+) cells in buffy coats. **B-E**: Data represent the mean ± SD of blood samples analyzed. Multiple unpaired *t* test. F-I: Data from 27 individuals are plotted. Spearman’s rank correlation test with correlation coefficient (*r*) and *p* values. Confidence intervals are calculated by linear regression analysis. **p ≤0.01; ***p≤0.001. Age: buffy coat: young: 18–23 yrs, *n* = 15, old: 60–70 yrs, *n* = 12; peripheral blood: young: 22–25 yrs, *n* = 5, old: 72–84 yrs, *n* = 5.

Aging is associated with massive changes in the composition of immune cells and immune responses. Elevated MDSC numbers are described in older adults with frailty and previous history of cancer [7]. Since both tumor development and aging can account for MDSC accumulation, MDSC numbers were analyzed in our study in healthy, non-frail older people, who were negatively diagnosed for cancer, acute infections, autoimmunity, neurodegenerative and severe cardiovascular diseases at the time point of blood sampling. Percentage of blood MDSCs and their subpopulations were defined in PBMCs isolated from fresh blood or buffy coats of healthy young and healthy old donors using flow cytometry according to Cassetta et al. and Bruderek at al. [3, 4] (Fig. 1A). Total MDSCs were stained as lin^neg^CD33^+^CD11b^+^HLA-DR^low/neg^. M-MDSCs are CD33^high^ CD11b^+^ HLA-DR^low/neg^ CD14^pos^ CD66^neg^ (G3 in Fig. 1A), while PMN-MDSCs are CD33^mid^ CD11b^+^ HLA-DR^low/neg^ CD14^neg^ CD66^pos^ (G5 in Fig. 1A). E-MDSCs are CD33^med^ CD11b^+^ HLA-DR^low/neg^ CD14^neg^ CD66^neg^ CD123^neg^ (G7 in Fig. 1A). Total MDSCs, M-MDSCs and PMN-MDSCs were significantly increased in percentage and cell number in the blood of old donors compared to young donors, while e-MDSCs were fairly detectable and reduced with age (Fig. 1B, C). Analysis of PBMCs derived from buffy coats showed similar results, although increase in PMN-MDSCs was not significant (Fig. 1D, E). Increased age correlated significantly with elevated blood proportions of total MDSCs (Fig. 1F) and M-MDSCs (Fig. 1G), but not significantly of PMN-MDSCs (Fig. 1H). Increase of MDSCs is accompanied by a decrease in lineage positive cells (B- T- NK- cells) (Fig. 1I). Since both fresh blood- and buffy coat-isolated MDSCs accumulate with age, we performed part of the experiments with buffy coat blood due to better availability.

### CD14 cells of old donors exhibit higher differentiation rate into MDSCs with increased suppressive activity

**Fig. 2:**
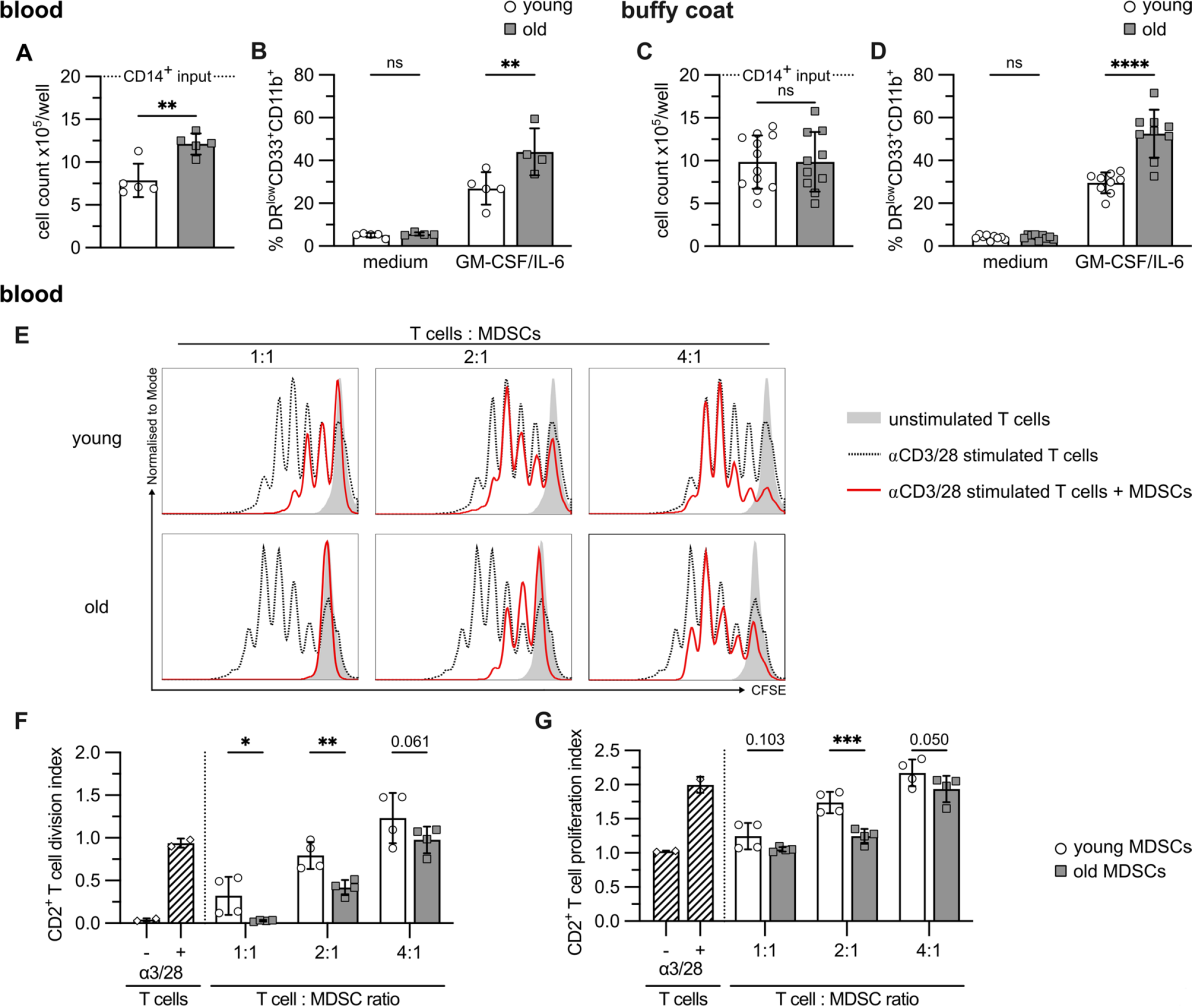
Blood-derived MDSCs of old donors exhibit increased immunosuppressive activity. **A-D**: CD14 cells isolated from peripheral blood (**A, B**) or buffy coat (**C, D**) of young and old donors were seeded with 2 × 10^6^ cells in the presence of GM-CSF and IL-6 or left untreated (medium). After 5 days of differentiation, cell numbers were determined (**A, C**) and percentage of CD33^+^CD11^+^HLA-DR^low^ cells was defined by flow cytometry (**B, D**). **E-G**: CFSE-labeled T cells were stimulated with plate-bound CD3/CD28 antibodies or left untreated. MDSCs were added at different T cell: MDSC ratios. After 5 days cells were stained for CD2 and T cell proliferation was defined by CFSE dilution by flow cytometry. E: representative flow cytometry histogram. Division (**F**) and proliferation (**G**) index of CD2 cells were calculated. **A-D**: Data represent the mean ± SD/samples analyzed. **A**: young *n* = 5, old *n* = 5; **B**: young *n* = 5, old *n* = 4, **C**: young: *n* = 12, old: *n* = 11; **D**: young *n* = 9, old *n* = 9 F, **G**: Data represent the mean ± SD of technical triplicates of each donor analyzed per group young *n* = 4, old *n* = 4. A, C: Unpaired *t* test, **B, D, F, G**: two-way ANOVA. *p ≤0.05; **p ≤0.01; ***p≤0.001; ****p≤0.0001; ns = non-significant. Age: peripheral blood: young: 22–25 yrs, old: 72–84 yrs; buffy coats: young: 18–23 yrs, old: 61–70 yrs; buffy coat donor for T cell isolation: young: < 25 yrs.

Next, we defined whether young and old MDSCs differ in their suppressive capacity. Deep functional analysis of peripheral blood (PB) MDSCs in healthy individuals is challenging due to low cell numbers and limited blood access. Therefore, we used an established protocol to generate M-MDSCs from isolated CD14 cells derived from peripheral blood (PB-MDSCs) or buffy coats (BC-MDSCs) [23, 24]. 2 × 10^6^ CD14 cells isolated from peripheral blood (Fig. 2A) or buffy coats (Fig. 2C) of young and old donors were differentiated with GM-CSF and IL-6. At day 5, at the end of the differentiation process, cultures from old donors contained more cells, although differences were not significant for buffy coat cultures. MDSC differentiation rate defined by the induction of CD33^+^CD11^+^HLA-DR^low^ cells (Supplementary Fig. 1A) was significantly higher if CD14 cells were isolated from old donors, independent of whether peripheral blood or buffy coats were used (Fig. 2B, D). Isolated CD14 cells left unstimulated served as control. To test whether age also affects the suppressive activity of PB- or BC-MDSCs, CFSE-labeled T cells were stimulated with plate-coated CD3/CD28 antibodies in the presence of decreasing numbers of MDSCs. When comparing inhibitory effects of MDSCs from young and old donors on T cell proliferation, T cells were always derived from the same young donor. T cell proliferation was defined by flow cytometry (Fig. 2E) and T cell division (Fig. 2F) and proliferation index (Fig. 2G) was calculated. Figure 2E, F and G depict the effect of MDSCs on the total CD2^+^ T cell population. Anti-CD3/28-induced proliferation was more efficiently inhibited by PB-MDSCs of old donors compared to PB-MDSCs derived from young donors. Of note, PB-MDSCs mostly affected the T cell division index. T cell division was significantly inhibited at T cell: MDSC ratios of 1:1 and 2:1, while MDSCs lost their inhibitory effects at a ratio of 4:1. Therefore, the following experiments were perfomed at T cell: MDSC ratios of 1:1 or 2:1. Surprisingly, MDSCs generated from old donors inhibited CD4^+^ T cells more efficiently than CD8^+^ T cells compared to MDSCs induced from young donors (Supplementary Fig. 1B). Similar results were obtained with BC-MDSCs, as those from older donors suppressed T cells stronger than those from younger donors (Supplementary Fig. 1C). Although T cells and BC-MDSCs were derived from different donors, allogeneic BC-MDSCs did not stimulate T cell proliferation (Supplementary Fig. 1D). In summary, these data show that blood-derived MDSCs from CD14 cells of old donors have an increased potential to differentiate into MDSCs with higher immunosuppressive capacity, indicating that age-dependent changes in hematopoiesis might contribute to differences in MDSC induction and function.

### Blood-derived MDSCs suppress primarily by direct cell contact

**Fig. 3:**
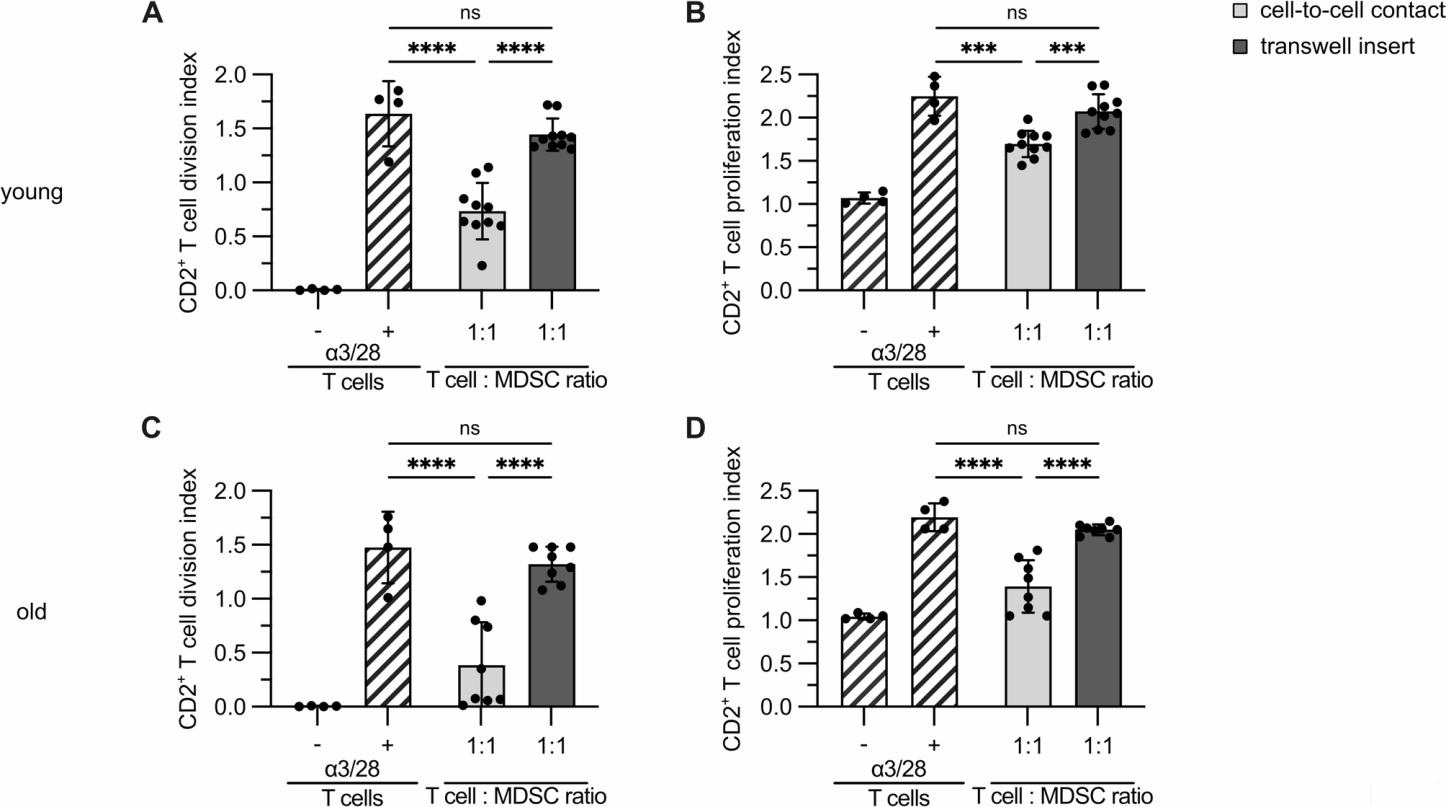
Blood-derived MDSCs prevent T cell proliferation by cell-to-cell contact. **A-D**: CD14 cells were isolated from buffy coats of young and old donors and stimulated with GM-CSF and IL-6 to generate MDSCs. CFSE-labeled T cells were stimulated with plate-bound CD3/CD28 antibodies. BC-MDSCs generated either from young (**A, B**) or old (**C, D**) donors were added directly to the wells or to the insert of a transwell at a T cell: MDSC ratio of 1:1. 5 days later, T cells were stained for CD2 and proliferation was determined by CFSE dilution by flow cytometry. Division (**A, C**) and proliferation (**B, D**) index was calculated. Data represent the mean ± SD of 10 proliferation assays performed with young MDSCs and 8 performed with old MDSCs. A-D: one-way ANOVA. ***p≤0.001; ****p≤0.0001; ns = non-significant. Age: T cell donors: 19–22 yrs, *n* = 5, MDSCs: young: 20–23 yrs, *n* = 5, old: 62–70 yrs, *n* = 4.

Suppression of T cell proliferation can be mediated by direct MDSC– T cell contact or by soluble factors. Therefore, we stimulated CFSE-labeled T cells with plate-bound CD3/CD28 antibodies. BC-MDSCs from either young (Fig. 3A, B) or old (Fig. 3C, D) donors were added at a ratio of T cell: MDSC of 1:1 directly to the well or into the transwell insert. T cell proliferation was measured after 5 days and the T cell division (Fig. 3A, C) and proliferation (Fig. 3B, D) index was determined. T cell division and proliferation index were significantly reduced if MDSCs had direct cell contact with T cells, while soluble factors derived from MDSCs in the transwell insert did not prevent T cell proliferation. Both young and old MDSCs reduced T cell proliferation only after direct co-culture, showing that independent of donor age MDSCs need to interact with T cells directly to prevent T cell expansion.

### Serum factors from young and old donors support the induction of MDSCs, but only serum from old donors increase the immunosuppressive capacity

**Fig. 4:**
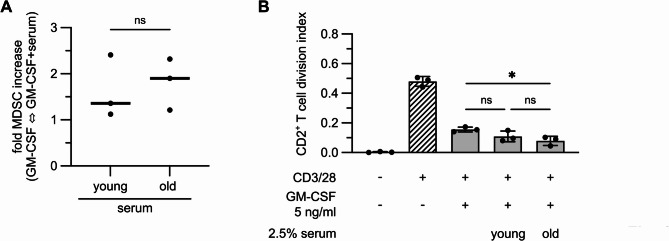
Serum factors boost MDSC induction and serum from old donors improves suppressive capacity of BC-MDSCs. **A, B**: CD14 cells derived from buffy coats of young donors were stimulated either with 5ng/ml GM-CSF alone or in the presence of 2.5% serum, which was pooled from either 5 young or 5 old donors. **A**: MDSC induction by GM-CSF alone or in the presence of serum from young and old donors was determined by analyzing the numbers of CD33^+^CD11^+^HLA-DR^low^ cells by flow cytometry. Fold increase compared to GM-CSF alone was calculated. **B**: MDSCs induced by GM-CSF alone or in the presence of pooled serum from young and old donors were incubated with CD3/28-activated CFSE-labeled T cells isolated from a young donor at a ratio T cell: MDSC = 4:1. After 5 days, cells were stained for CD2 and T cell proliferation was defined by CFSE dilution by flow cytometry and division index was calculated. **A**: Data represent the mean ± SD of fold increase in MDSC induction compared to GM-CSF-only induced MDSCs. Student-*t*-test. **B**: Data represent the mean ± SD of 3 proliferation assays performed with MDSCs derived from 3 different donors. **A**: students t-test; **B**: one-way ANOVA. *p≤0.05; ns = non-significant. Age: CD14 cell donors: 19–24 yrs, *n* = 3, serum: young: 21–25 yrs, *n* = 5, old: 84–93 yrs, *n* = 5; T cell donor: 23 yrs.

Apart from age-associated genetic alterations in hematopoietic progenitors, MDSC accumulation in old donors might be promoted by inflammaging, reflected by changes in serum factors. Therefore, CD14 cells derived from buffy coats of young donors were induced for MDSC differentiation by GM-CSF and 2.5% serum. In comparison to the former experiments, GM-CSF was used at a lower concentration of 5 ng/ml to prevent that GM-CSF activation masks the serum effect. Serum factors were defined for 14 old and 15 young donors. Surprisingly, from the analyzed factors (IL-1β, IL-1RA, IL-4, -6, -10, -18, CRP, TNFα, S100A8/9, IFNγ, TGF-β) only IL-10 and S100A8/9 were increased in the serum of old donors, while TGF-β serum concentrations were significantly higher in the serum of young donors (Supplementary Fig. 2). To exclude effects of individual sera on MDSC differentiation, serum from 5 young and 5 old donors was chosen randomly, pooled and used for activation of BC-derived CD14 cells from young donors in the presence of GM-CSF (5ng/ml). After 5 days of culture, serum-induced MDSCs were harvested and extensively washed to remove remaining serum. Induction rate of MDSCs was increased by young and old serum compared to GM-CSF treatment alone with a slightly better induction rate by old serum (Fig. 4A). Next, serum-induced BC-MDSCs were co-incubated with anti-CD3/28 activated T cells at a ratio of 4:1. BC-MDSCs induced by young and old serum significantly reduced T cell division, however, only MDSCs generated from old serum could further decrease T cell division rates compared to GM-CSF only-induced MDSCs (Fig. 4B). In summary, we could show that serum factors of both young and old donors promote MDSC induction and expansion. Only MDSC induced in the presence of old serum, however, exhibited increased immunosuppressive capacity, indicating that inflammaging supports immunosuppressive functions of MDSCs.

### Old MDSCs skew T cell responses towards type 2 T cell immunity

**Fig. 5:**
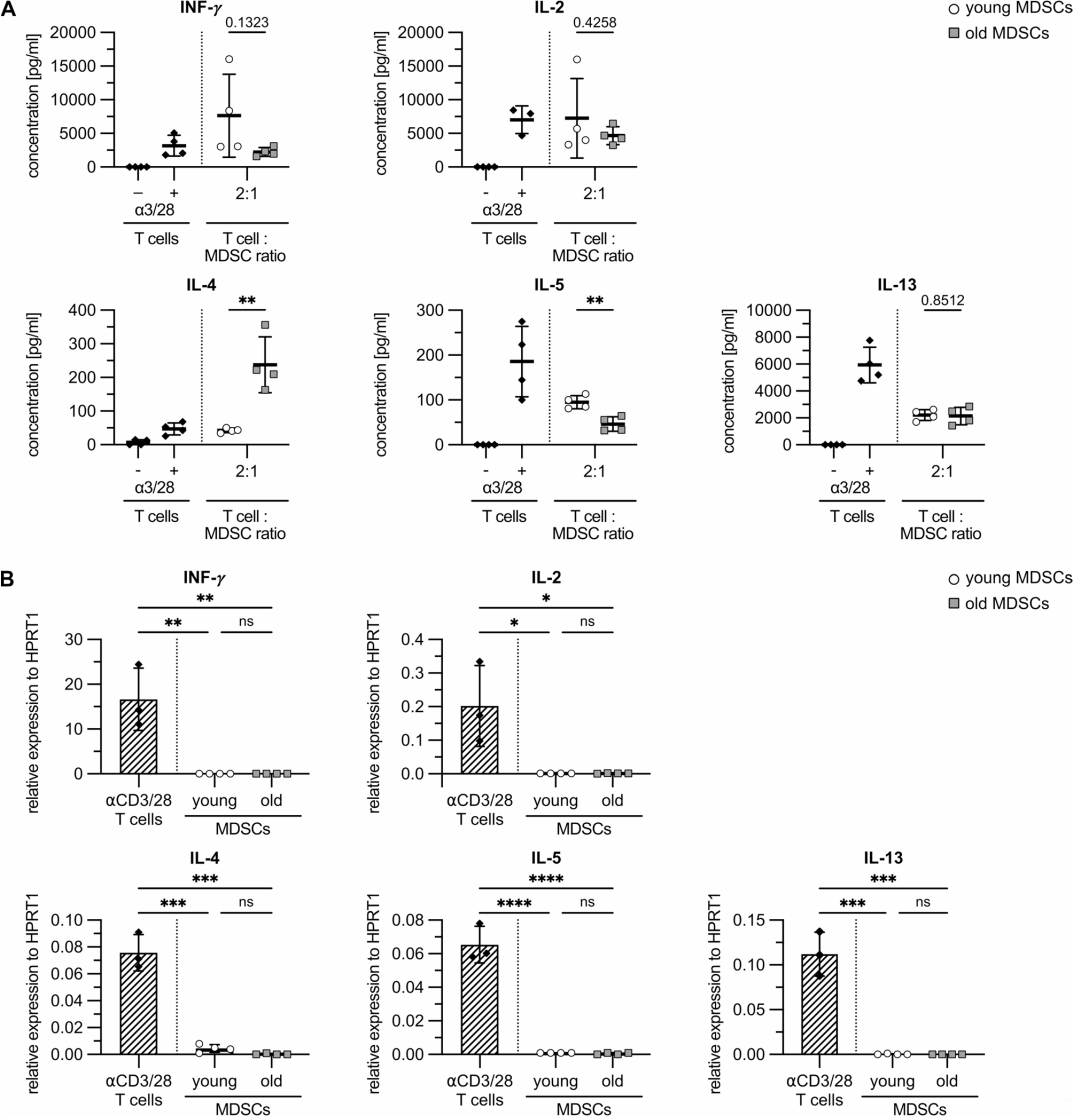
PB-MDSCs derived from old donors polarize immune response towards Th2 immunity. **A, B**: CD14 cells isolated from peripheral blood of young and old donors were differentiated to MDSCs by GM-CSF and IL-6. **A**: PB-MDSCs were added at a T cell: MDSCs ratio of 2:1 to anti-CD3/28 activated T cells. T cells alone were stimulated with anti-CD3/28 to control cytokine induction in the absence of MDSCs. Culture supernatants were collected at day 4 and analyzed for cytokine expression. **B**: PB-MDSCs used in the proliferation assay were analyzed by qRT-PCR for corresponding cytokine expression. Relative expression of cytokines to HPRT1 was calculated. **A**: Cytokine concentrations represent the mean ± SD of T cell proliferation assays performed in the presence of PB-MDSCs derived from 4 young and 4 old donors. Cytokine values of T cells alone represent the mean of two experiments performed in duplicates. **B**: Data represent the mean ± SD of biological triplicates from PB-MDSCs derived from 4 different young and old donors or from 3 T cell donors. **A**: unpaired t-test; **B**: Two-way ANOVA. ****p≤0.0001; ***p≤0.001; **p≤0.01; *p≤0.05; ns = non-significant. Age: T cells: < 25 yrs, MDSCs: young: 22–25 old: 72–84 yrs.

As aging proceeds, T cells as the main interacting partner of MDSCs, also undergo age-related changes. One feature is the decline in the Th1/Th2 balance [25–27]. Th2 skewing might either reflect the history of antigen contact or might be promoted by increased MDSC numbers, which are associated with Th2 immunity in different disease entities [28–30]. Therefore, supernatant of CD3/CD28-stimulated CFSE-labeled T cells co-cultured at a ratio of 2:1 with young and old PB-MDSCs was analyzed for Th1 and Th2 cytokines at day 4 (Fig. 5A). Unstimulated and anti-CD3/28-activated T cells served as negative and positive control. Th1 cytokine IFN-γ was slightly but not significantly decreased in the presence of old PB-MDSCs, while IL-2 production was indistinguishable in cultures with young or old PB-MDSCs. Of note, IL-4 as the master Th2 cytokine, which is indispensable for Th2 differentiation, was only significantly elevated in the presence of PB-MDSCs derived from old donors and was even 5 times higher than in CD3/28-activated T cells. IL-13 and IL-5 expression was reduced 3–5 times in MDSC co-cultures compared to CD3/28-activated T cells. IL-5 production was lower in presence of PB-MDSCs from old donors compared to PB-MDSCs from young donors. RT-PCR analysis for IFN-γ, IL-2, − 4, -5, and − 13 showed that none of the cytokines are expressed on RNA level in PB-MDSCs independent of donor age, clearly indicating that T cells are the cellular source of cytokine production (Fig. 5B). Thus, only blood-derived MDSCs from old donors polarize immune responses towards Th2 immunity and may contribute to the age-dependent decline in Th1/Th2 balance.

### Old MDSCs exhibit metabolic differences

**Fig. 6:**
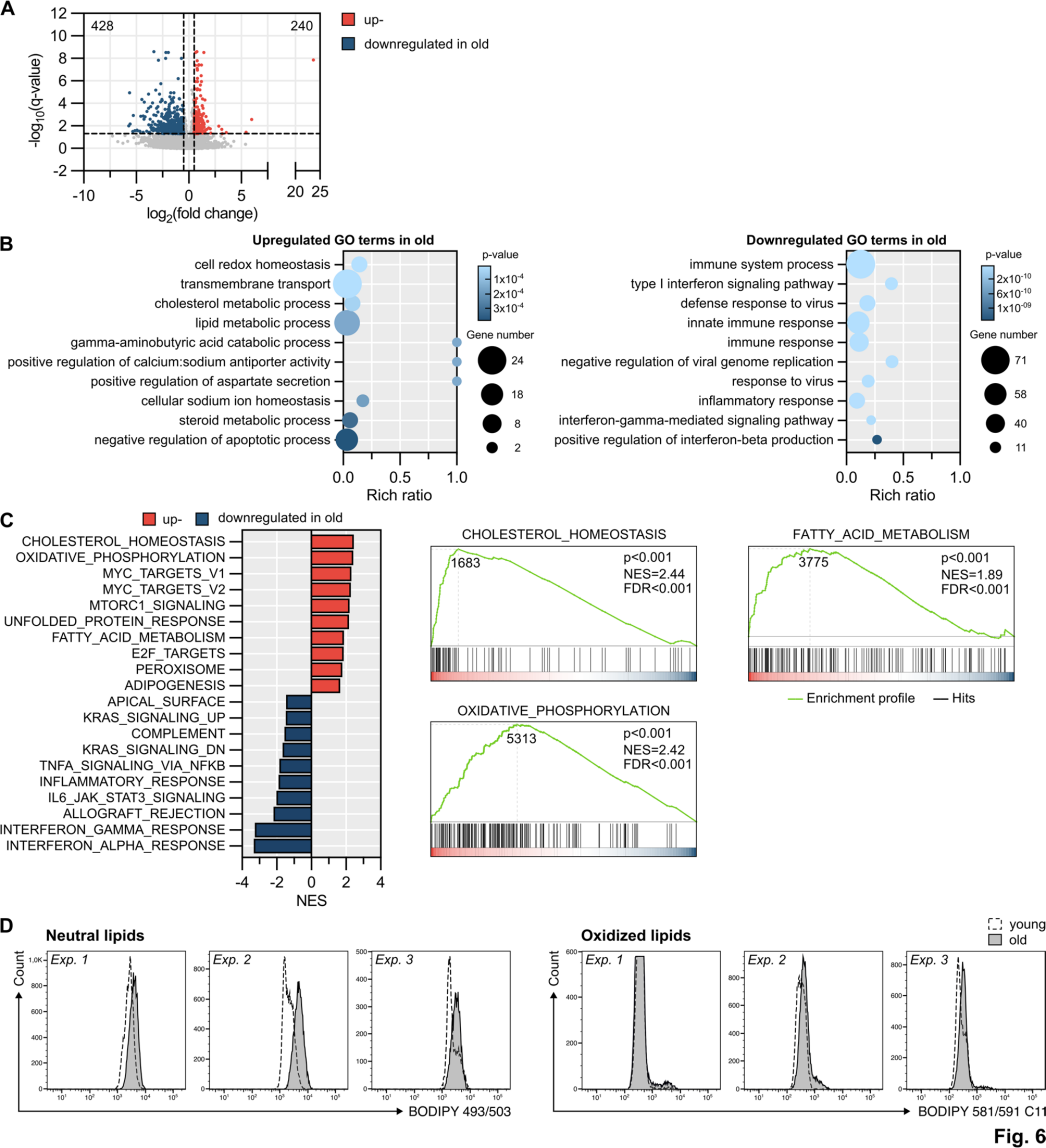
RNA-seq analysis of MDSCs from young and old donors. Peripheral blood derived CD14 cells from young and old donors were differentiated into MDSCs using GM-CSF and IL-6, followed by RNA sequencing. **A**: Volcano plot showing up- and downregulated differentially expressed genes (DESeq2 package:|log2(FC)| ≥ 0.5; q < 0.05). **B**: Gene Ontology (GO) biological process analysis of up- and downregulated DEGs. **C**: Gene Set Enrichment Analysis (GSEA) with corresponding enrichment graphs (Hallmark gene set; NES > 1 or < -1; *p* < 0.05; FDR < 0.25). **D**: Buffy coat derived CD14 cells from young and old donors were differentiated into MDSCs using GM-CSF and IL-6, followed by staining for neutral (BODIBY 493/503) and oxidized (BODIBY 581/591 C11) lipids. Flow cytometry histograms show three independent experiments, each comparing a young and an old donor. PB-MDSCs: Age: young: 23–25 yrs, *n* = 5, old: 72–84 yrs, *n* = 4, with outliner removed *n* = 3; BC-MDSCs: Age: 18–23 yrs, *n* = 3, old: 70–74 yrs, *n* = 3.

Next, we analyzed whether functional differences of young and old blood-derived MDSCs can be attributed to transcriptomic differences by RNA-seq. Only 225 genes were differentially expressed if CD14 cells of young and old donors are compared (Supplementary Fig. 3A). Pathways associated with increased innate immune responses and a decreased response of antigen processing (Supplementary Fig. 3B) were differently expressed between the two groups. During MDSC differentiation, CD14 cells of young and old donors underwent an excessive change in their transcriptome, depicted by about 8000 genes differentially expressed in PB-MDSCs compared to CD14 cells isolated either from young (Supplementary Fig. 4A) or old donors (Supplementary Fig. 5A). GO terms and GSEA analysis indicated that transcriptomic changes during the development from CD14 cells to MDSCs cells were similar in young and old donors (Supplementary Fig. 4B, C and 5B, C). Despite a comparable differentiation process, young and old PB-MDSCs differed in a total of 688 genes from which 240 genes were up-regulated and 428 genes were down-regulated in old MDSCs with a log2 fold change of 0.5 (Fig. 6A). GO enrichment analysis indicated that old MDSCs exhibited upregulation of metabolic processes such as cholesterol homeostasis, oxidative phosphorylation and fatty acid metabolism. Genes involved in immune system processes and responses, however, were down-regulated (Fig. 6B). GSEA analysis confirmed that genes involved in oxidative phosphorylation, cholesterol homeostasis and fatty acid metabolism are enriched in old MDSCs, while immune cell activities involving genes mediating IFN-γ or -α responses or IL-6-Jak-STAT3 signaling were down-regulated (Fig. 6C). Pathways involved in cholesterol homeostasis and lipid metabolism exhibited up-regulation of many DEGs (Supplementary Fig. 6). Since cholesterol metabolism and lipid overload in MDSCs correlates with their immunosuppressive function, we compared the intracellular content of neutral (BODIPY 493/503) and oxidized lipids (BODIPY581/591 C11) of BC-MDSCs from young and old donors (Fig. 6D). Increased amount of neutral lipid droplets was detected in all MDSCs from old donors, while two donors also exhibited increased expression of oxidized lipids, strongly indicating that increased immunosuppressive activity is partially mediated by elevated lipid content. In summary, these data show that differentiation of CD14 cells into MDSCs activated similar gene expression profiles independent of the donor age. However, blood-derived MDSC from old donors significantly increase their metabolic activity in pathways such as cholesterol synthesis, fatty acid oxidation or lipid metabolism which are all known to improve immunosuppressive function of MDSCs.

## Discussion

MDSC accumulation is associated with any kind of inflammation ranging from tumor development, infection and trauma to pregnancy, obesity and stress. Especially in the tumor setting, the role of MDSCs is a predictive factor for disease severity, metastasis development, overall survival and a main driver of impaired anti-tumor immunity. The impact of MDSCs, however, in the development of immunosenescence is less specified because aging is mostly accompanied by disease development which makes it difficult to distinguish between MDSC induction due to age or age-related diseases. In the current study, we compared induction and functions of MDSCs in the peripheral blood of non-frail old donors, which were negatively diagnosed for cancer, autoimmunity, neurodegenerative- and severe cardiovascular diseases and acute infections with young adults. To our knowledge, we show here for the first time that MDSCs increase during aging independent of diagnosed age-associated diseases at the time of analysis. Interestingly, not only the increase in MDSC numbers is specific for old individuals, but also functional changes, including increased inhibition of T cell functions, polarization towards type 2 immunity and metabolic changes. Thus, MDSC aging might strongly contribute to immunosenescence of cellular immunity.

Analysis of PBMCs isolated either from peripheral blood or buffy coats of young (18–25 yrs) or old (61–84 yrs) donors showed a continuous increase in MDSC numbers with age. Increase in MDSC numbers was less pronounced in buffy coats compared to peripheral blood, since blood donation is only allowed until 70 yrs and average age of buffy coat donors is lower than from donors of peripheral blood. This might also explain why both PMN-MDSCs and M-MDSCs are significantly elevated in peripheral blood of old donors, but only M-MDSC numbers are increased in old buffy coat donors. Surprisingly, Alves et al. reported a significant predominance of PMN-MDSCs in individuals older than 80 yrs, while the M-MDSC subtype was statistically higher in young individuals [8]. Since no information about donor health status was given, a comparison with our study is difficult. The positive association of frailty and a history of cancer on MDSC numbers is clearly indicated in the early work by Verschoor et al., which, however, did not further specify the expansion of different MDSC subpopulations [7].

Collectively, all these studies indicate an age-dependent increase of MDSCs, but do not give any indications about functional changes in MDSCs during aging. The work by Magri et al. showed that MDSCs differentiated from GM-CSF/G-CSF stimulated BM-derived progenitors of young and old donors exhibited identical suppressive capacity, but reduced MDSC-induction capacity [18]. This is in contrast to our findings where we showed that MDSCs derived from CD14 cells isolated either from peripheral blood or buffy coats of old individuals inhibited both the percentage of dividing T cells after CD3/28 activation and the average number of divisions more efficiently than MDSCs derived from young donors. Likewise, old CD14 cells showed a better MDSC-induction capacity in vitro than young CD14 cells.

We are fully aware that in vitro-generated CD14-derived MDSCs might exhibit different characteristics and functions than in vivo-induced MDSCs. However, since strong disease-derived triggers of MDSC induction are missing in our study cohort, MDSCs are present only at very low percentages in the blood and ethical restrictions make it nearly impossible to obtain sufficient numbers of MDSCs for functional assays from healthy donors. Additionally, long-lasting sample preparations such as MDSC sorting or magnetic bead isolation in order to obtain isolated MDSCs comes to the expense of viability and functionality. Likewise, cryopreservation changes the distribution of MDSC subsets [31–33]. Nevertheless, in vitro-generated human MDSCs induced either from total PBMCs, blood-derived CD14 cells or hematopoietic progenitors by different combinations of growth factors have been shown to acquire immunosuppressive functions comparable to ex vivo-induced MDSCs [34–37]: e.g. MDSCs induced from CD14 cells by human melanoma cells resemble MDSCs characterized in patients with advanced stage melanoma [38]. Likewise, the mechanisms of immunosuppression are similar in blood-derived MDSCs and ex vivo-induced MDSCs. In vivo-induced MDSCs suppress T or NK cell functions preferentially by direct cell-cell contact [39–41] and blood-derived MDSCs also exhibit their immunosuppressive function by direct interaction with T cells. As well, old PB-MDSCs exhibited upregulation of metabolic processes including cholesterol homeostasis, fatty acid and lipid metabolism. Content of neutral lipids is increased in old BC-MDSCs compared to young BC-MDSCs, which was in most of the analyzed samples associated with an increase in oxidized lipids. Likewise, expression of fatty acid transport proteins, elevated fatty acid uptake and oxidation are linked to improved immunosuppressive functions in ex vivo analyzed tumor-infiltrating MDSCs [42–45]. Likewise, in our study, only the function of M-MDSCs was analyzed due to the lack of protocols that efficiently induce PMN-MDSCs.

Age-related functional changes in MDSCs do not only affect T cell expansion but also T cell polarization. In the presence of old MDSCs, T cells were shifted towards Th2 immunity with increased IL-4 and decreased IFN-γ secretion. IL-4 production of T cells in the presence of MDSCs was even higher than IL-4 produced in T cells after CD3/28-activation. Th2 cytokines IL-5 and − 13 were also secreted, however, they were not elevated in the presence of MDSCs from old donors, indicating non-coordinated Th2 cytokine expression and heterogeneity in the Th2 producing cells. Importantly, MDSCs themselves are no Th2 cytokine producers, although we cannot exclude that in co-culture conditions MDSCs are triggered to produce Th2-specific cytokines. How MDSCs induce Th2 polarization in the absence of T cell-derived Th2 cytokine production is currently not clarified. Possibly, expression of co-stimulatory molecules and transcription factors in MDSCs might promote Th2 cell stimulating signals corresponding to DC subsets specialized for Th2 priming [46]. MDSC-induced Th2 polarization was recently also reported in sepsis, pregnancy or after allogeneic bone marrow transplantation and traumatic lung injury [28–30, 47]. Furthermore, IL-4 and IL-15 are essential for the differentiation of virtual memory T cells (T_VM_), which accumulate in mice and humans during aging [48, 49]. T_VM_ exhibit a phenotype similar to conventional antigen-experienced memory T cells, although they never encountered antigen. T_VM_ lost their proliferative response to TCR stimulation and acquire features of senescence [50, 51].

Low-grade inflammation and myeloid skewing might be the main drivers of age-related MDSC accumulation and functional changes. Old blood donors had modestly increased serum levels of IL-10 and S100A8/9. IL-10 and S100A8/9 are inducers of MDSC expansion and activation and at the same time act as immunosuppressive factors secreted by MDSCs themselves [52–54]. Surprisingly, TGF-β as another major immunosuppressive MDSC-derived cytokine, was not elevated in old donors, indicating that elevated MDSC levels do not simultaneously account for increased serum concentrations of TGF-β. However, this is not surprising, as TGF-β is not exclusively produced by MDSCs, but also by all types of immune cells and various tissue cells. Likewise, we could show that, at least in vitro, the inhibition of T cell proliferation requires direct contact with MDSCs, whereas MDSC-derived soluble factors only play a minor role for inhibition of T cell proliferation. MDSC-induction rate from CD14 cells was only marginal increased by serum from old donors compared to serum from young donors. However, addition of serum from old donors to the MDSC induction cultures ameliorated the immunosuppressive potential compared to cultures induced only in presence of GM-CSF. This improvement was not observed if serum from young donors was added. Besides inflammaging, the age-related bias in myeloid cell production also seems to be involved in MDSC accumulation. First, CD14 cells of old donors are more prone to develop into MDSCs with an increased immunosuppressive potential and secondly, transcriptomic profiles of CD14 cells and PB-MDSCs differ dependent of donor age.

Although the data clearly indicate that aging of MDSCs affects T cell function, a deeper understanding of the interconnection between aging in MDSCs and cellular immunity and the mutual influence of other immunoregulatory cells, such as T_regs_, B_regs_ or NK T cells, with MDSCs, is required to define, whether MDSCs represent suitable targets for supporting healthy aging.

## Conclusions

Our results show increased MDSC numbers in the blood of old “healthy” donors, which are negatively diagnosed for MDSC-driving diseases indicating that aging per se contributes to MDSC expansion. Transcriptome analysis showed major changes in metabolism of blood-derived MDSCs from young and old donors, which correlate with their effect on T cell functions. MDSCs derived from old donors inhibited CD3/28-induced T cell proliferation more efficiently than MDSCs from young donors and skewed T cells towards Th2 immunity, clearly showing that MDSCs strongly contribute to age-related impairment of cellular immunity. Only serum factors from old donors further improved immunosuppressivity of MDSCs indicating inflammaging as a driver for age-related changes in MDSCs. Altogether, these data strongly indicate that aging of innate immunity involves MDSCs expansion and functional changes which directly relate to impairment of T cell immunity and inefficient immune responses in old individuals.

## Electronic supplementary material

Below is the link to the electronic supplementary material.

Supplementary Material 1

## Data Availability

The datasets used and / or analyzed during the current study are available from the corresponding author on reasonable request. Raw and processed RNA-seq data have been deposited in the NCBI Gene Expression Omnibus (GEO) under accession number GSE279455.

## Abbreviations

BC: Buffy coat
BC-MDSCs: MDSCs derived from CD14 cells isolated from buffy coats
BM: Bone marrow
cDNA: Complementary DNA
CMV: Cytomegalovirus
DEGs: Differentially expressed genes
EBV: Epstein-barr-virus
EDTA: Ethylene diamine tetraacetic acid
GSEA: Gene set enrichment analysis
GO: Gene ontology
HPRT1: Hypoxanthine phosphoribosyltransferase 1
HSPC: Hematopoietic stem and progenitor cell
HSV: Herpes simplex virus
IL: Interleukin
MDSC: Myeloid-derived suppressor cell
M-MDSC: Monocytic myeloid-derived suppressor cell
PB: Peripheral blood
PB-MDSCs: MDSCs derived from CD14 cells isolated from peripheral blood
PBMC: Peripheral blood mononuclear cell
PMN-MDSC: Polymorphonuclear myeloid-derived suppressor cell
PGE_2_: Prostaglandin E_2_
TCR: T-cell receptor
TGF: Transforming growth factor
TNF: Tumor necrosis factor
VEGF: Vascular endothelial growth factor
yrs: Years
