# Supplementary material for: Aging modulates the immunosuppressive, polarizing and metabolic functions of blood-derived myeloid-derived suppressor cells (MDSCs)
Source: Immun Ageing. 2025 Jul 8;22:29. doi: 10.1186/s12979-025-00524-w (PMC12235767; doi:10.1186/s12979-025-00524-w)
Supplement: Supplementary file 1 — Supplementary Material 1 [file 12979_2025_524_MOESM1_ESM.docx]

**Supplementary Material 1:**

**1. Supplementary Tables**

**Supplementary Table 1: List of antibodies used in flow cytometry**

| **Target** | **Fluorochrome** | **Supplier** | **Clone** | **Order #** |
| --- | --- | --- | --- | --- |
| CD11b | APC | Invitrogen by ThermoFisher Scientific | ICRF44 | 17-0118-42 |
| CD11b | APC/Cy7 | BioLegend Inc. | ICRF44 | 301341 |
| CD14 | Pacific Blue | BD Pharmingen™ by BD | M5E2 | 558121 |
| CD14 | PerCP/Cy5.5 | BioLegend Inc. | HCD14 | 325621 |
| CD15 | FITC | BD Pharmingen™ by BD | HI98 | 555401 |
| CD16 | PE-Cy7 | BD Pharmingen™ by BD | 3G8 | 560716 |
| CD19 | Brilliant Violet 421 | BioLegend Inc. | H1B19 | 302233 |
| CD2 | APC | Invitrogen by ThermoFisher Scientific | RPA-2.10 | 17-0029-42 |
| CD20 | Brilliant Violet 421 | BioLegend Inc. | 2H7 | 302329 |
| CD3 | Brilliant Violet 421 | BioLegend Inc. | SK7 | 344833 |
| CD3 | Pacific Blue | BD Pharmingen™ by BD | UCHT1 | 558117 |
| CD33 | PE | Invitrogen by ThermoFisher Scientific | WM-53 | 12-0338-42 |
| CD4 | Pacific Blue | BD Pharmingen™ by BD | RPA-T4 | 558116 |
| CD56 | Brilliant Violet 421 | BioLegend Inc. | HCD56 | 318327 |
| CD66b | FITC | Bio-Rad Laboratories Inc. | 80H3 | MCA216F |
| CD8a | PE-Cy7 | Invitrogen by ThermoFisher Scientific | RPA-T8 | 25-0088-42 |
| HLA-DR | APC-eFlour780 | Invitrogen by ThermoFisher Scientific | LN3 | 47-9956-42 |
| HLA-DR | APC | BD Pharmingen™ by BD | G46-6 | 560896 |

**Supplementary Table 2: List of primers**

| **Target** | **Sequence** | **Product length** |
| --- | --- | --- |
| HPRT1 | 5‘ - GAGATGGGAGGCCATCACATTGTAGCCCTC - 3‘ | 193 |
|  | 5‘ - CTCCACCAATTACTTTTATGTCCCCTGTTGACTGGTC - 3‘ |  |
| IFNγ | 5‘ - TCGGTAACTGACTTGAATGTCCA - 3‘ | 93 |
|  | 5‘ - TCGCTTCCCTGTTTTAGCTGC - 3‘ |  |
| IL-2 | 5‘ - TCCTGTCTTGCATTGCACTAAG - 3‘ | 161 |
|  | 5‘ - CATCCTGGTGAGTTTGGGATTC- 3‘ |  |
| IL-4 | 5‘ - CCAACTGCTTCCCCCTCTG - 3‘ | 150 |
|  | 5‘ - TCTGTTACGGTCAACTCGGTG - 3‘ |  |
| IL-5 | 5‘ - TCTACTCATCGAACTCTGCTGA - 3‘ | 132 |
|  | 5‘ - CCCTTGCACAGTTTGACTCTC - 3‘ |  |
| IL-13 | 5‘ - GAGGATGCTGAGCGGATTCTG - 3‘ | 85 |
|  | 5‘ - CACCTCGATTTTGGTGTCTCG - 3‘ |  |

**2. Supplementary Figures**

**
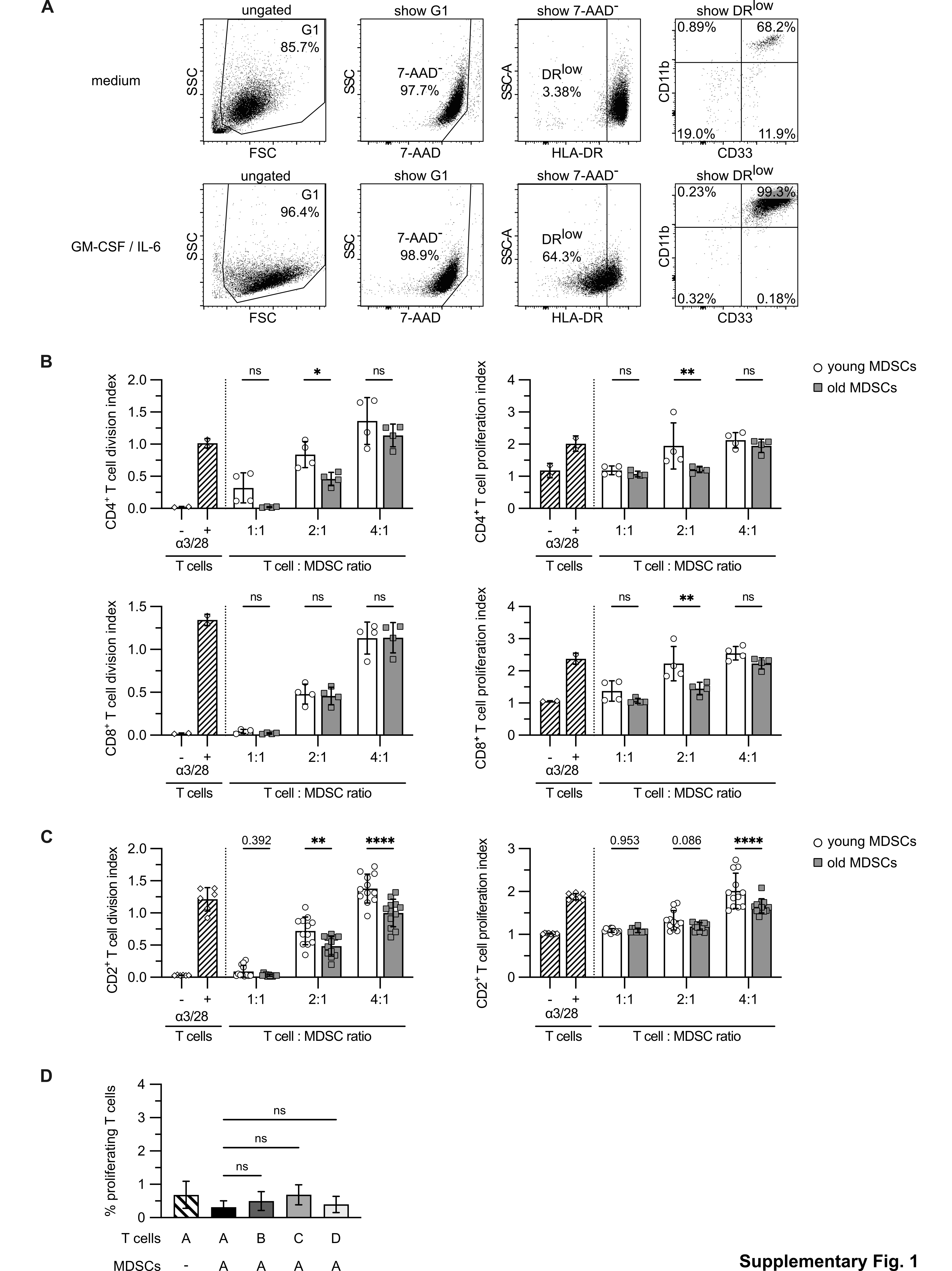
**

**Supplementary Figure 1: Blood-derived MDSCs from old donors exhibit increased immunosuppressive capacity and do not activate allogeneic T cells.** A-D: CD14 cells isolated from buffy coats (A, C, D) or peripheral blood (B) were differentiated by GM-CSF (20 ng/ml) and IL-6 (20 ng/ml) to MDSCs for 5 days or left untreated (medium). A: representative FACS staining and gating strategy. B, C: CFSE-labeled allogeneic T cells were stimulated in the presence or absence of plate-bound CD3/CD28 antibodies. MDSCs were added at different T cell : MDSC ratios. After 5 days, cells were stained for CD2, CD4 and CD8 and T cell proliferation was defined by CFSE dilution by flow cytometry. Division and proliferation index of CD4^+^ and CD8^+^ T cells were calculated in the presence of PB-MDSCs (B). Division and proliferation index of CD2 cells were calculated in the presence of BC-MDSCs (C). Age: peripheral blood: young: 22-25 yrs, (n=4); old: 72-84 yrs, (n=4); buffy coats: young: 18-23 yrs, (n=12); old: 61-70 yrs, (n=12); buffy coat donor for T cell isolation: young: < 25 yrs. D: MDSCs generated from donor A were cocultured with CFSE-labeled T cells from autologous donor A or allogeneic donors B, C and D. After 5 days cells were stained for CD2 and T cell proliferation was determined by CFSE dilution by flow cytometry. Age: T cells 30-52 yrs; MDSCs: 30 yrs. B, C: Data represent the mean ± SD of technical triplicates of individuals analyzed per group. B, C: two-way ANOVA, D: one-way ANOVA. *p ≤0.05; **p ≤0.01; ****p≤0.0001; ns=non-significant.

**
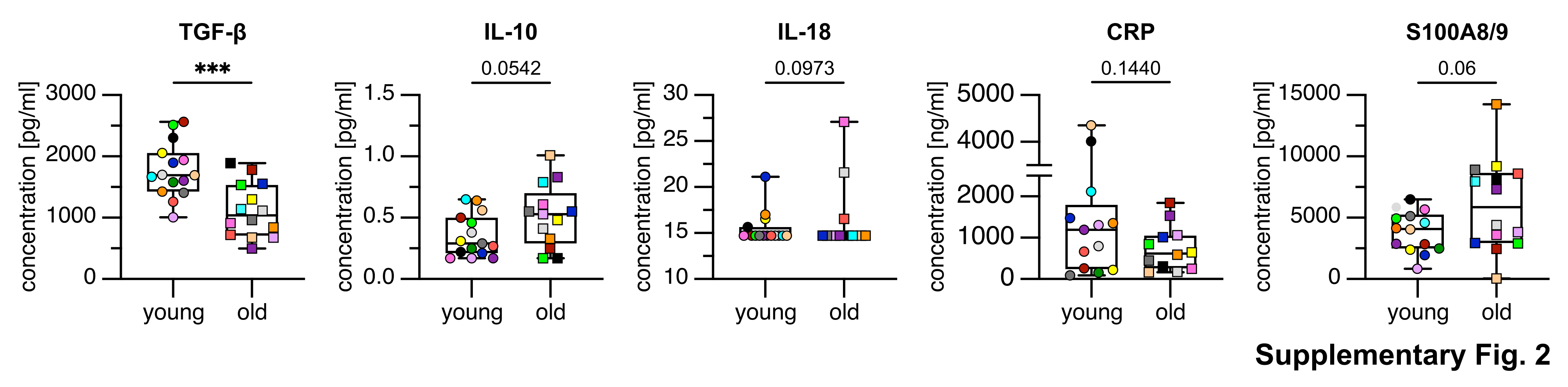
**

**Supplementary Figure 2: Serum factors in young and old donors.** Human serum samples from healthy young and old donors were analyzed for the indicated factors by ProcartaPlex Multiplex Immunoassay. Each color represents one individual/group. From the analyzed factors (IL-1β, IL-1RA, IL-4, -6, -10, -18, CRP, TNFα, S100A8/9, IFNγ, TGF-β) only TGF-β, IL-10, CRP, IL-18 and S100A8/9 were above the detection level of the assay. If values of TGF-β, IL-10, CRP, IL-18 and S100A8/9 were out of range for single donors, the values were either set to the minimal or maximal value measurable by the assay. Data represent the mean ± SD of individuals / group. Young: 21-29 yrs, n=15; Old: 78-93 yrs, n=14. Unpaired student’s t-test (for normally distributed samples) and Mann-Whitney test (for not normally distributed samples). ***p≤0.001.

**
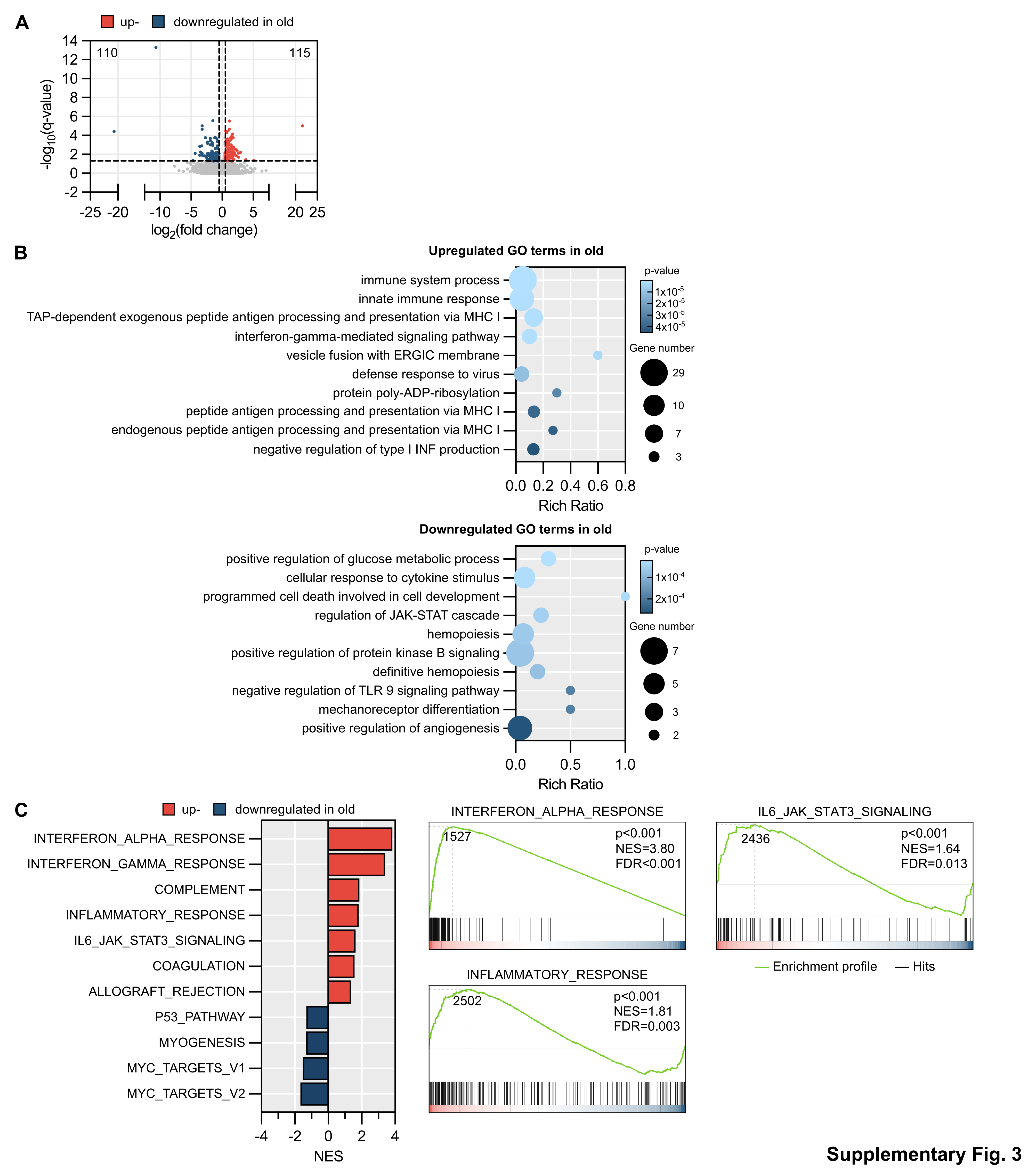
**

**Supplementary Figure 3: Transcriptomic differences in CD14 cells derived from peripheral blood of young and old donors.** RNA sequencing was performed on peripheral blood-derived CD14 cells from young and old donors. A: Volcano plot showing up- and downregulated differentially expressed genes (DESeq2 package: |log2(FC)| ≥ 0.5; q < 0.05). B: Gene Ontology (GO) biological processes analysis of up- and downregulated DEGs. C: Gene Set Enrichment Analysis (GSEA) with corresponding enrichment graphs (Hallmark gene set; NES > 1 or < -1; p < 0.05; FDR < 0.25). CD14: young: 23-25 yrs, n=4; old: 72-84 yrs, n=3.

**
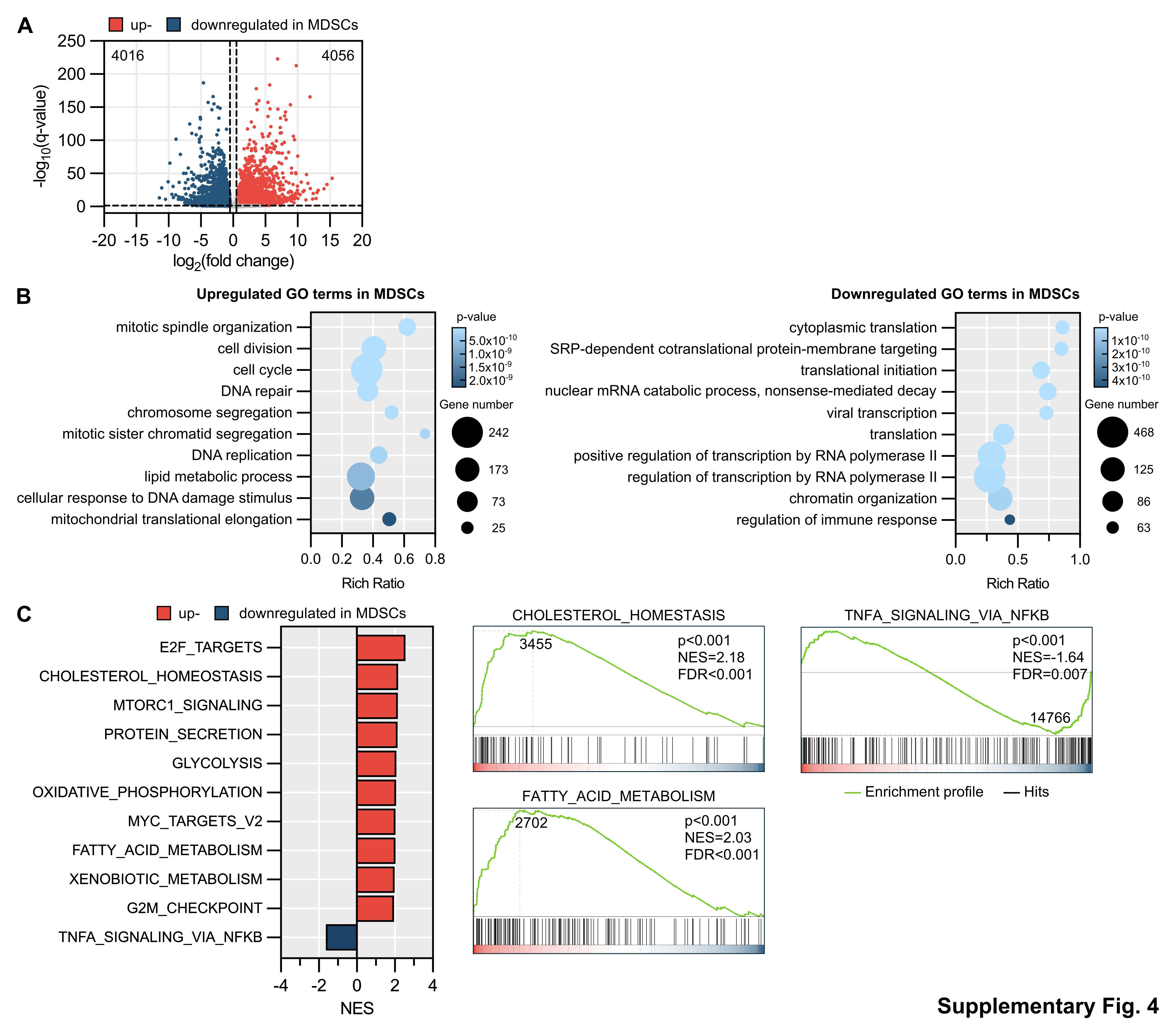
**

**Supplementary Figure 4: Transcriptomic differences between CD14 cells and PB-MDSCs derived from young donors.** Peripheral blood-derived CD14 cells were isolated from young donors and differentiated into MDSCs using GM-CSF and IL-6. RNA sequencing was performed on CD14 cells and MDSCs. A: Volcano plot showing up- and downregulated differentially expressed genes (DESeq2 package: |log2(FC)| ≥ 0.5; q < 0.05). B: Gene Ontology (GO) biological processes analysis of up- and downregulated DEGs. C: Gene Set Enrichment Analysis (GSEA) with corresponding enrichment graphs (Hallmark gene set; NES > 1 or < -1; p < 0.05; FDR < 0.25). Age: 23-25 yrs. CD14: n=4. MDSCs: n=5.


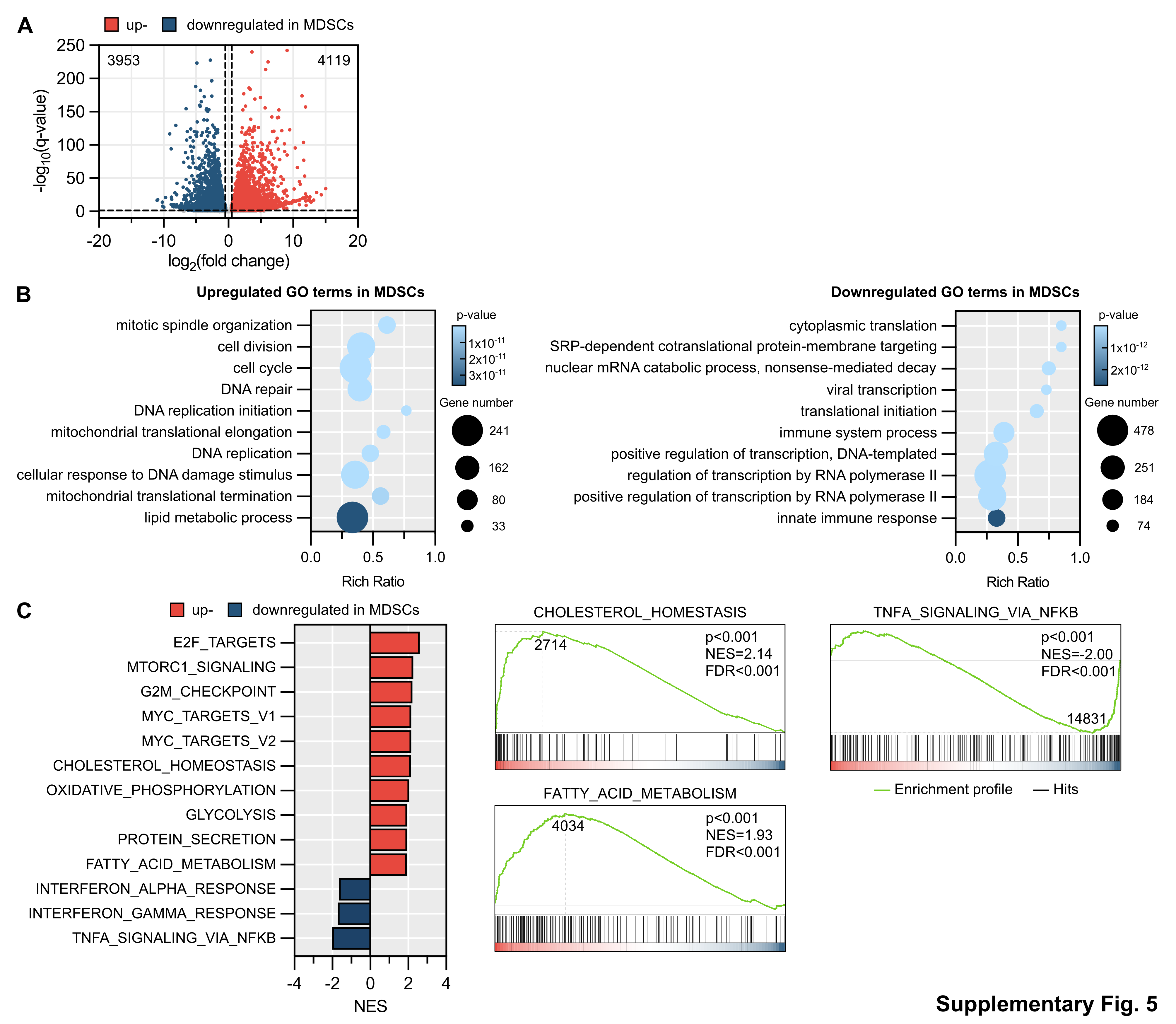


**Supplementary Figure 5: Transcriptomic differences between CD14 cells and PB-MDSCs derived from old donors.** Blood-derived CD14 cells were isolated from old donors and differentiated into MDSCs using GM-CSF and IL-6. RNA sequencing was performed on CD14 cells and MDSCs. A: Sample correlation heatmap displaying the Pearson’s correlation coefficients. B: Principal component analysis (PCA) based on the top variable genes. C: Volcano plot showing up- and downregulated differentially expressed genes (DESeq2 package: |log2(FC)| ≥ 0.5; q < 0.05). D: Gene Ontology (GO) biological processes analysis of up- and downregulated DEGs. E: Gene Set Enrichment Analysis (GSEA) with corresponding enrichment graphs (Hallmark gene set; NES > 1 or < -1; p < 0.05; FDR < 0.25). Age: 72-84 yrs. CD14: n=3. MDSCs: n=4, with outliner removed n=3.

**
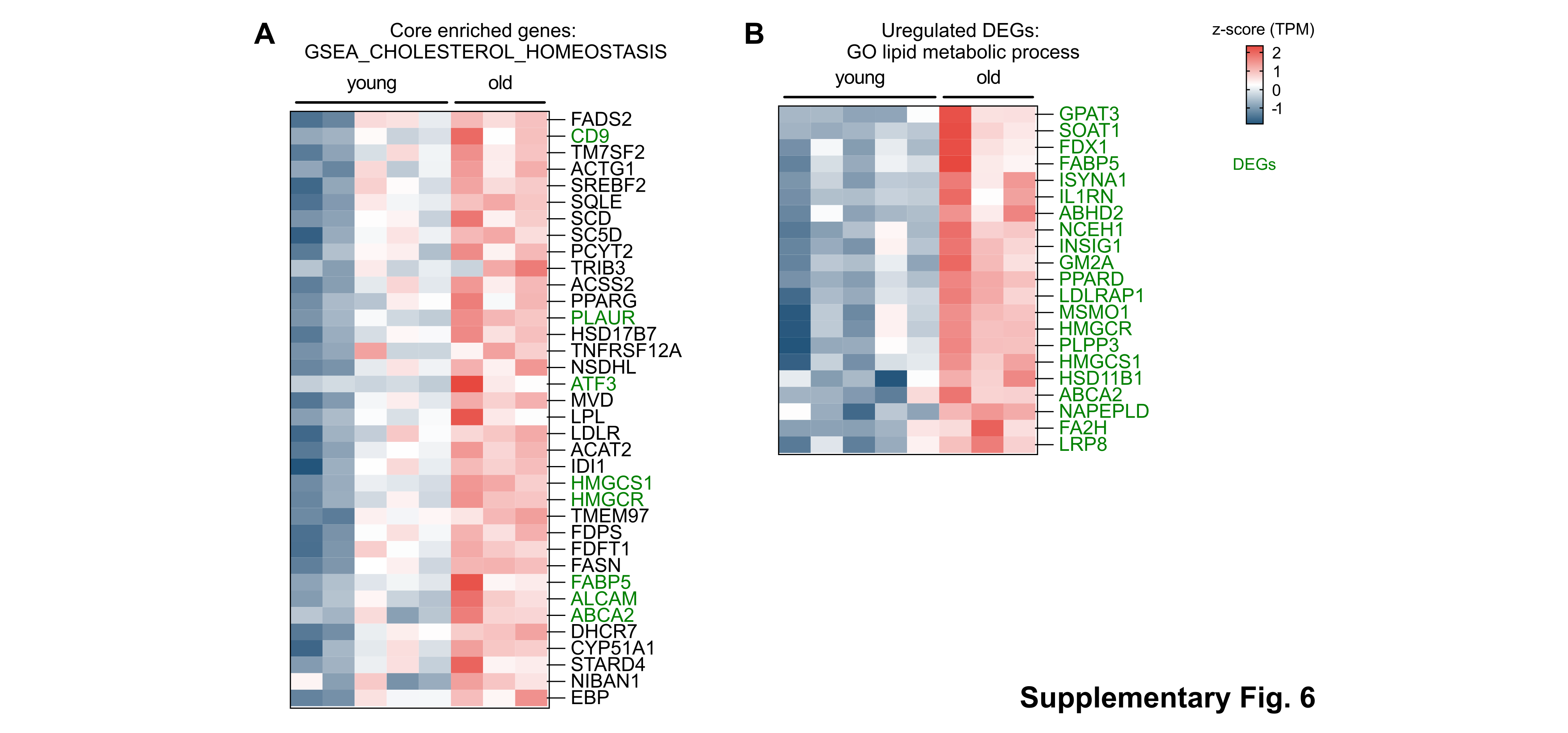
**

**Supplementary Figure 6: DEGs in cholesterol homeostasis and lipid metabolism in PB-MDSCs.** Peripheral blood derived CD14 cells from young and old donors were differentiated into MDSCs using GM-CSF and IL-6, followed by RNA sequencing. A: Gene Set Enrichment Analysis (GSEA) with corresponding heatmap of core enriched genes in the Hallmark gene set cholesterol homeostasis, with DEGs highlighted (Hallmark gene set; NES > 1 or < -1; p < 0.05; FDR < 0.25). B: Gene Ontology (GO) biological process analysis of up-regulated DEGs (DESeq2 package: |log2(FC)| ≥ 0.5; q < 0.05) with corresponding gene heatmap of lipid metabolic process. Age: young: 23-25 yrs, n=5; old: 72-84 yrs, n=4, with outliner removed n=3.
